# Advanced Sensing Technology in Structural Health Monitoring

**DOI:** 10.3390/s26175438

**Published:** 2026-08-28

**Authors:** Giulio D’Emilia, Emanuela Natale

**Affiliations:** Department of Industrial and Information Engineering and Economics (DIIIE), University of L’Aquila, 67100 L’Aquila, Italy

## 1. Introduction

Structural Health Monitoring (SHM) has become an increasingly important research area in engineering, driven by the increasing demand for safer, more reliable, and sustainable civil structures and infrastructure, as well as industrial systems. The ability to assess the structural integrity of civil and industrial systems throughout their service life is fundamental to preventing catastrophic failures, optimizing maintenance strategies, extending service life, and reducing life-cycle costs [1]. In recent years, the traditional paradigm of scheduled inspections has evolved toward condition-based and predictive maintenance, in which decisions are supported by continuous measurements and data-driven assessments of structural conditions [2].

Remarkable advances in sensing technologies have enabled this evolution. Modern SHM systems are no longer limited to the deployment of individual sensors but increasingly rely on integrated measurement systems capable of acquiring large amounts of heterogeneous data with high spatial and temporal resolution. Ultrasonic guided-wave techniques, piezoelectric transducers, fiber-optic sensors, optical measurement techniques, electromagnetic sensors, and inertial devices are examples of sensing technologies that have enabled increasingly detailed and reliable information acquisition on structural behavior, while improving measurement accuracy, robustness, and operational flexibility [2]. At the same time, advances in electronics, embedded systems, communication technologies, and wireless sensing have enabled the implementation of distributed sensing architectures capable of operating under real-world conditions [3].

The increasing availability of measurement data has simultaneously shifted the focus from sensor development alone toward the extraction of meaningful information from complex datasets. Signal processing techniques have consequently become an essential component of modern SHM systems, enabling the identification of subtle changes in structural behavior that would otherwise remain undetectable [4]. More recently, artificial intelligence techniques have emerged as powerful tools for supporting damage detection, classification, and prognosis. Data-driven approaches are increasingly complementing traditional physics-based models, offering new opportunities for handling highly nonlinear problems and large-scale monitoring applications while improving the automation of decision-making processes [5,6].

Despite these significant advances, several scientific and technological challenges remain. The design of reliable sensing systems requires careful consideration of measurement uncertainty, sensor positioning, environmental variability, long-term stability, and data quality [7]. Similarly, the successful implementation of intelligent monitoring systems depends not only on the performance of learning algorithms but also on the quality, representativeness, and interpretability of experimental measurements. Consequently, metrology continues to play a fundamental role in ensuring that the combination of sensing technologies and data processing algorithms provides reliable information for structural assessment and maintenance planning [7,8].

Another characteristic of contemporary SHM research is its inherently multidisciplinary nature. Similar sensing principles can be successfully applied to remarkably different domains, spanning aerospace, civil, and industrial applications. Although these systems differ significantly in terms of materials, loading conditions, deterioration mechanisms, and operational environments, they share common methodological challenges concerning sensor system design, experimental measurements, signal interpretation, and damage characterization. This convergence has stimulated the development of innovative sensing strategies that can often be transferred across different engineering disciplines.

The papers collected in this Special Issue, “Advanced Sensing Technology in Structural Health Monitoring”, provide a representative overview of these ongoing developments. The published contributions address complementary aspects of modern SHM, including ultrasonic guided-wave inspection, optical metrology, electromagnetic sensing, long-term structural monitoring, machine learning-based damage detection, and multidisciplinary approaches for assessing civil infrastructure and cultural heritage. Together, they demonstrate how recent advances in sensing technologies, measurement methodologies, and intelligent data analysis are contributing to more reliable, accurate, and versatile SHM systems. The diversity of the application fields represented in this collection further highlights the increasingly interdisciplinary character of SHM and emphasizes the central role that advanced sensing technologies will continue to play in the future evolution of the field.

## 2. Overview of Published Articles

The nine contributions collected in this Special Issue illustrate the broad range of sensing technologies, measurement approaches, and data analysis methods currently being investigated for SHM. Although the studies address different materials, structures, and application contexts, they share the common objective of extracting reliable information on structural condition from measurements.

Several contributions investigate piezoelectric sensing, including the generation and detection of ultrasonic guided waves, highlighting different aspects and ways in which these technologies can be integrated into SHM systems.

Aleksiewicz-Drab et al. (1) investigate the directivity and excitability of ultrasonic shear waves generated by piezoceramic transducers through numerical modeling and experimental investigations. Their results provide insights into how transducer configuration influences wave generation, with implications for the design and optimization of ultrasonic guided-wave inspection systems.

Batista et al. (2) present an FPGA-based intelligent SHM system for composite aerospace structures, using piezoelectric sensors and Lamb waves to detect, classify, and locate damage in real time. The system combines signal processing techniques, including power spectral density, wavelet transform, and principal component analysis, with support vector machines for damage classification and Mahalanobis distance for outlier management. This integration enables sensing, signal processing, and intelligent damage assessment to be performed within the monitoring system, supporting real-time operation.

Xu et al. (3) investigate the flexural behavior and damage evolution of reinforced concrete beams subjected to explosive loading using piezoelectric smart aggregates embedded in the beams. The internal damage is monitored through the Wavelet Packet Energy Analysis method, from which an internal crack development index is derived and related to the residual flexural capacity. The results show a significant reduction in the flexural capacity of the pre-damaged beam and reveal the evolution of internal cracking during subsequent loading up to failure.

A further application of piezoelectric sensing is presented by Nicassio et al. (4), who measure the vibrational response of aerospace structures subjected to impacts and use machine learning models to estimate impact energy and identify damaged areas. The study combines experimental sensing with data-driven analysis for impact characterization and damage detection.

Other contributions explore sensing principles based on optical and electromagnetic phenomena.

Chiominto et al. (5) propose a measurement technique using a polarization camera to identify fiber orientation in carbon fiber components, as this parameter can significantly affect their strength and stiffness, and thus their structural integrity. Particular attention is devoted to the metrological characterization of the method, including assessments of repeatability, measurement uncertainty, and the effects of acquisition conditions.

Radhakrishnan et al. (6) investigate the use of a planar monopole antenna for crack detection in metallic structures. The presence and propagation of cracks, introduced as a perturbation in the antenna’s metallic ground plane, produce a shift in the resonance frequency, which is used as an indicator of structural damage. The approach is investigated through finite element simulations and experimentally validated for different crack dimensions and orientations.

The Special Issue also highlights the use of machine learning and deep learning techniques to transform measured signals into information on structural condition.

As discussed above, the work by Batista et al. (2) demonstrates the integration of machine learning with embedded signal processing, while Nicassio et al. (4) employ supervised machine learning models to extract information on impact events and structural damage from vibration data.

Ullah et al. (7) propose a leak detection and classification framework for multi-fluid pipelines based on acoustic emission signals. Their approach combines Continuous Wavelet Transform and Savitzky–Golay filtering to generate enhanced time-frequency scalograms, which are then analyzed using a CNN and a lightweight Vision Transformer. The results demonstrate how advanced signal representations and deep learning architectures can be combined to achieve accurate classification while maintaining computational efficiency.

Finally, two contributions demonstrate the application of advanced measurement approaches in complex, long-term monitoring scenarios.

Machelski et al. (8) investigate long-term changes in the geometry and internal forces of a steel cable-stayed bridge by combining multi-year geodetic surveys with SHM data. In the study, the geometric changes measured by geodetic surveys are introduced into a finite element model to estimate the corresponding changes in stay forces, which are then compared with the forces measured by load cells over an eight-year monitoring period. The study illustrates the value of continuous measurements for understanding structural behavior over extended periods and supporting maintenance-related decisions.

Casula et al. (9) address the assessment of ancient monuments through a multidisciplinary approach that integrates non-destructive 3D geomatic techniques, including terrestrial laser scanning and close-range photogrammetry, with ultrasonic and electrical resistivity methods, and complementary petrographic analyses. The combined approach enables high-precision modeling of both surfaces and internal features and supports the identification and characterization of deterioration caused by environmental and anthropogenic factors.

Taken together, the contributions demonstrate that the integration of different sensing principles, rigorous measurement methodologies, advanced signal processing, and intelligent data analysis increasingly characterizes contemporary SHM. The variety of approaches and application domains represented in the Special Issue also highlights the importance of selecting and combining sensing and data analysis strategies according to the characteristics of the structure, the damage mechanisms of interest, and the requirements of the monitoring application.

## 3. Conclusions

The contributions collected in this Special Issue clearly illustrate the rapid evolution of Structural Health Monitoring toward increasingly intelligent, integrated, and application-oriented measurement systems. While advanced sensing technologies remain the foundation of every monitoring strategy, the papers demonstrate that their effectiveness increasingly depends on the integration of accurate measurements with robust data processing. This synergy enables more reliable damage detection, improved structural assessment, and more informed maintenance decisions across a wide range of engineering applications.

A common feature emerging from the published papers is the remarkable diversity of sensing principles currently available for SHM. Ultrasonic guided-wave techniques, piezoelectric transducers, optical polarization techniques, microwave sensing, and multidisciplinary non-destructive testing methods each address different monitoring requirements, confirming that no single sensing technology is universally applicable. Instead, the selection of the most appropriate measurement strategy depends on the characteristics of the monitored structure, the expected damage mechanisms, and the operational environment.

Another important aspect highlighted by the Special Issue is the increasing role of data-driven methodologies. Artificial intelligence techniques are no longer considered merely post-processing tools but have become integral components of modern sensing systems, enabling the automated interpretation of measurement data and supporting real-time decision-making. At the same time, the contributions emphasize that the effectiveness of these approaches ultimately depends on the quality and reliability of the measurements acquired, reaffirming the central role of metrology in SHM.

Finally, the papers demonstrate the multidisciplinary nature of contemporary SHM research, encompassing applications in aerospace, civil and industrial systems, as well as cultural heritage. Despite the diversity of these application domains, they share common scientific challenges related to sensing technologies, experimental measurements, data interpretation, and structural assessment. The Guest Editors hope that the contributions presented in this Special Issue will stimulate further research on advanced sensing technologies and foster new interdisciplinary collaborations aimed at developing increasingly reliable, intelligent, and sustainable Structural Health Monitoring systems.
